# Impact of chemical structure of flavanol monomers and condensed tannins on *in vitro* anthelmintic activity against bovine nematodes

**DOI:** 10.1017/S0031182015001912

**Published:** 2016-02-18

**Authors:** OLIVIER DESRUES, CHRISTOS FRYGANAS, HONORATA M. ROPIAK, IRENE MUELLER-HARVEY, HEIDI L. ENEMARK, STIG M. THAMSBORG

**Affiliations:** 1 Parasitology and Aquatic Diseases, Department of Veterinary Disease Biology, University of Copenhagen, Dyrlægevej 100, DK-1870 Frederiksberg C, Denmark; 2 Chemistry and Biochemistry Laboratory, School of Agriculture, Policy and Development, University of Reading, Reading RG6 6AT, UK; 3 Section for Bacteriology, Pathology and Parasitology, National Veterinary Institute, Technical University of Denmark, Frederiksberg, Denmark; 4 Section for Parasitology, Department of Laboratory Services, Norwegian Veterinary Institute, PO Box 750 Sentrum, N-0106 Oslo, Norway

**Keywords:** proanthocyanidins, ruminant, cattle, *Ostertagia ostertagi*, *Cooperia oncophora*, larval feeding inhibition, motility assay

## Abstract

Plants containing condensed tannins (CT) may have potential to control gastrointestinal nematodes (GIN) of cattle. The aim was to investigate the anthelmintic activities of four flavan-3-ols, two galloyl derivatives and 14 purified CT fractions, and to define which structural features of CT determine the anti-parasitic effects against the main cattle nematodes. We used *in vitro* tests targeting L1 larvae (feeding inhibition assay) and adults (motility assay) of *Ostertagia ostertagi* and *Cooperia oncophora*. In the larval feeding inhibition assay, *O. ostertagi* L1 were significantly more susceptible to all CT fractions than *C. oncophora* L1. The mean degree of polymerization of CT (i.e. average size) was the most important structural parameter: large CT reduced larval feeding more than small CT. The flavan-3-ols of prodelphinidin (PD)-type tannins had a stronger negative influence on parasite activity than the stereochemistry, i.e. *cis- vs trans*-configurations, or the presence of a gallate group. In contrast, for *C. oncophora* high reductions in the motility of larvae and adult worms were strongly related with a higher percentage of PDs within the CT fractions while there was no effect of size. Overall, the size and the percentage of PDs within CT seemed to be the most important parameters that influence anti-parasitic activity.

## INTRODUCTION

The potential of nutraceutical plants for the sustainable control of gastrointestinal nematodes (GIN) in ruminant livestock is still an under-explored area (Hoste *et al.* 2015). Their use could forestall the emergence of GIN resistance to available anthelmintic drugs and reduce the substantial economic losses due to these pathogens. Plants produce a wide range of secondary metabolites mainly to protect themselves from diseases and herbivores. These plant secondary metabolites include condensed tannins (CT; *syn.* proanthocyanidins), which are polyphenols that are able to bind proteins and other molecules such as polysaccharides, lipids, as well as metal ions (Schofield *et al.* 2001; Jakobek, 2015). CT are polymers that consist of various flavan-3-ol units which are defined by their hydroxylation, stereochemistry and substitution patterns. They tend to occur not as single CT molecules but as complex CT mixtures in plants. Therefore, these polymeric mixtures are described in terms of average polymer lengths [or mean degree of polymerization (mDP)] and as molar percentages of their flavan-3-ol subunits; the most common subunits are catechin and epicatechin [which are found in procyanidin (PC)-type tannins] and gallocatechin and epigallocatechin [which are found in prodelphinidin (PD)-type tannins]. Their stereochemistry is defined on the basis of *cis*-flavan-3-ols (epicatechin and epigallocatechin) and *trans*-flavan-3-ols (catechin and gallocatechin) (Williams *et al.* 2014*a*). Moreover, the attachment of substituents such as galloyl groups can also occur in CT (Spencer *et al.* 2007). This structural diversity affects protein-binding affinity as well as the biological activity of CT, as recently shown for *in vitro* ruminal methane production and fermentation (Saminathan *et al.* 2014, 2015; Hatew *et al.* 2015). Therefore it is important to assess how not only the tannin concentration, but also the tannin structure influence anthelmintic properties against different life cycle stages of the most important GIN species. A dose-dependency for CT has been demonstrated for small ruminant and cattle nematodes (Brunet *et al.* 2007; Novobilský *et al.* 2013; Molan, 2014). Studies until now have demonstrated that PD subunits were more potent than PC subunits in inhibiting the motility and exsheathment of third-stage larvae (L3) of *Haemonchus contortus* in sheep (Brunet and Hoste, 2006), motility of L3 of *Ascaris suum* of pigs (Williams *et al.* 2014*a*) or hatching of eggs of *Trichostrongylus colubriformis* of sheep (Molan *et al.* 2003). The galloylation of flavan-3-ols has also been shown to enhance the anthelmintic activity against L3 in ruminants (Molan *et al.* 2003; Brunet and Hoste, 2006). The CT subunits are commercially available as pure flavan-3-ols and CT that differ in subunit composition can be extracted and purified from various plant sources. This approach is much faster than attempting to separate the different CT groups from a single plant source, such as sainfoin or *Lotus* sp., where they occur as complex mixtures. This approach can also be used to overcome constraints in CT structures imposed by biosynthesis, especially for investigating differences in *cis*/*trans*-stereochemistry, which varies greatly between plant species (Porter, 1988). Such contrasting CT fractions are needed to give an insight into which structural features should be targeted by plant selection or breeding before recommending these bioactive compounds in livestock feeds. It has previously been reported that larger CT polymers in comparison with smaller ones more efficiently inhibit the feeding ability of first-stage larvae (L1) of *Ostertagia ostertagi* and *Cooperia oncophora* in cattle (Novobilský *et al.* 2013), and also the motility of L4 of *A. suum* and *Oesophagostomum dentatum* in pigs (Williams *et al.* 2014*a*, *b*). However, CT extracts and fractions tend to vary in terms of tannin contents and composition, which can complicate the interpretation of results compared to using pure compounds, e.g. flavan-3-ols. In fact, the CT contents in the earlier study of bovine nematodes ranged from 3 to 60 g CT/100 g fraction (Novobilský *et al.* 2013), and CT have been sparsely investigated in cattle as compared with small ruminants.

Thus, the aim of our study was to investigate the relation between CT structure and anti-parasitic activity using several commercial flavan-3-ols and galloyl derivatives, and highly purified CT fractions that covered a very wide range of structures. We applied *in vitro* tests to free-living (L1) and adult stages of the most important cattle nematodes, *O. ostertagi* and *C. oncophora.*

## MATERIALS AND METHODS

### Flavan-3-ol monomers and CT from plants

Four different flavan-3-ols and two galloyl derivatives were purchased from Sigma-Aldrich Ltd. (Denmark): catechin (C), epicatechin (EC), gallocatechin (GC), epigallocatechin (EGC), gallocatechin gallate (GCg) and epigallocatechin gallate (EGCg). CT previously described and tested *in vitro* against GIN in small ruminants (Quijada *et al.* 2015) from 14 European plants were used. Briefly, they included: bark and phloem from pine tree (*Pinus sylvestris*), above-ground sainfoin plants (*Onobrychis viciifolia*), pericarp from hazelnuts (*Corylus avellana*), leaves from blackcurrant collected from two sites (samples A and B; *Ribes nigrum*), flowers from white clover from two sites (samples A and B; *Trifolium repens*), flowers from *Tilia* spp., bark from willow (*Salix* spp.), leaves from walnut (*Juglans regia*), leaves from birch (*Betula* spp.), leaves and twigs from goat willow (*Salix caprea*) and weeping willow catkins (*Salix babylonica*). The details concerning the collection and drying procedures of plant materials, the extraction of tannins with acetone/water (7:3; v/v) and the purification of CT on Sephadex LH-20 columns yielding fractions F1 and F2 were described in Quijada *et al.* (2015). In the present study, we focused on the F2 fractions whereas only a few F1 fractions were included (goat willow leaves, pine bark and sainfoin). The CT fractions were degraded by thiolysis according to Quijada *et al.* (2015) prior to analysis by high-performance liquid chromatography as described by Williams *et al.* (2014*a*, *b*). This provided information on the CT content and composition in terms of mDP, molar percentages of PC- or PD-type subunits (PC/PD ratio) within CT and *cis*- or *trans*-flavan-3-ols within CT.

### Nematodes

Young naïve male calves were experimentally infected to propagate the different parasite species. The study was approved by the Animal Experiments Inspectorate, Ministry of Justice, Denmark (Ref. 2013-15-2934-00763). Care and maintenance of the calves were in accordance with applicable Danish and European guidelines. Inocula consisted of infective larvae (L3) of *O. ostertagi* and *C. oncophora* either mixed (assay with flavan-3-ol monomers; L3 recovered were 30% *O. ostertagi* and 70% *C. oncophora*) or separately (assays with CT fractions). Fresh feces was collected rectally during the patency period and L1 were prepared as previously described for cattle nematodes (Novobilský *et al.* 2011) and used for *in vitro* tests. Calves mono-infected with either *C. oncophora* or *O. ostertagi* were euthanized 28 and 38 days post-infection, respectively, and the adult worms were immediately recovered from the contents after embedding in agar and migration in warm saline water (2–3 h at 38 °C), according to Slotved *et al.* (1996).

### *In vitro* motility and feeding inhibition of first-stage larvae (L1)

The larval feeding inhibition assay (LFIA) including the preparation of the labelling of *Escherichia coli* with fluorescein isothiocyanate (FITC) as larval food source, was performed as described by Jackson and Hoste (2010). All tested compounds were serially diluted in phosphate buffer saline PBS (milliQ water; pH 6·9) at 3 concentrations, in triplicates, as previously used for LFIA with cattle nematodes (Novobilský *et al.* 2011). Briefly, 1300 *µ*L of either PBS as negative control, flavan-3-ol monomers at concentrations (10, 40 and 160 *µ*g mL^−1^) or CT fractions (2·5, 10 and 40 *µ*g of CT mL^−1^) were added to 1·5 mL Eppendorf tubes for each replicate. In the case of CT fractions, dilutions were adjusted for CT content as this parameter varied between 64 and 100%. Then, 100 *µ*L containing approximately 100 newly hatched larvae were added to each tube. After 2 h incubation at 25 °C, 10 *µ*L of FITC labelled *E. coli* was added. Subsequently, the tubes were horizontally incubated for 18 h at 25 °C, then centrifuged (6000 ***g***, 2 min) and 850 *µ*L of the supernatant was carefully removed. The remaining solution with larvae was placed in a counting chamber and read under a fluorescent microscope (blue filter 475–490 nm) at ×100 magnification. Fed larvae were differentiated according to Novobilský *et al.* (2011). Additionally, follow-up experiments were performed: (i) in order to ensure that the inhibitory effect of CT on larval feeding was directed against L1 and not due to an alteration of *E. coli* by CT, we modified the procedure by removing CT from two fractions that differed in their mDP values (samples A and B from blackcurrant leaves) and replaced them with control media before adding the bacterial food source. The modified assay was done in triplicate with *O. ostertagi* L1 and CT fractions were adjusted for CT contents; (ii) F1 fractions of sainfoin, goat willow leaves and pine bark were tested against *C. oncophora* L1 at 20 *µ*g of CT mL^−1^, to verify the lack of anthelmintic activity of sainfoin F1 previously found against pig nematodes (Williams *et al.* 2014*a*); (iii) the putative interference of impurities such as sucrose, which is the major sugar in sainfoin (Marais *et al.* 2000), was assessed in LFIA with *O. ostertagi* by pre-incubating the F2 fraction of sainfoin with or without sucrose (50:50; w/w) at 2·5, 10 and 40 *µ*g of CT mL^−1^; simultaneously (iv) the inhibition of *O. ostertagi* L1 motility was assessed with 8 CT fractions, where any movement within 5 s was counted as motile larvae.

### *In vitro* adult motility inhibition assay

First, adult worms were washed 3 times, following recovery by migration, with a warm solution of PBS (milliQ water; pH 7·2) containing penicillin/streptomycin (P4333, Sigma-Aldrich) at a concentration of 1:100 and amphotericin B (A2942, Sigma-Aldrich) at 1:250. Due to limited number of worms this assay was only performed with CT fractions of willow bark and blackcurrant leaves A and B. The CT fractions were diluted in the same control medium as described for the washing procedure, at 150 and 300 *µ*g mL^−1^ without adjustment for CT content, and transferred to 24-well plates in triplicates (1 mL per well). Then, approximately 5 active worms were selected and added to each well containing control media only (negative control) or diluted CT fractions. The motility of each worm was scored after 10 s observation as: (3) active movements; (2) slow movements; (1) moving only buccal or anterior parts; (0) non-motile; at 4, 6, 8, 20, 24 and 30 h of incubation at 38 °C.

### Statistical analyses

Statistical analyses were performed using R (version 3.2.0). In the LFIA with flavan-3-ol monomers, comparison of differences in larval feeding percentages was based on the three replicates including control values and assessed by multiple linear regression depending on the factors: flavan-3-ol monomers and dose, and their interaction. In the LFIA with CT fractions, the mean larval feeding percentage was calculated from the three replicates and was considered as the outcome in the multiple linear regression of explanatory variables: parasite species, dose (categorical), percentage of PD and *cis*-subunits, mDP and their interactions with the dose. Additionally, a model including CT subunit-percentages, i.e. C, EC, GC and EGC instead of percentage of PD and *cis*-subunits was also tested. For these models, the outcomes were square root transformed using the command ‘boxcox’ and multicollinearity in the model was identified with variance inflation factors (VIF). All final models were checked for normality with the Shapiro–Wilk test. The correlations between structural parameters of CT in fractions, mean percentages of inhibition in LFIA for the two nematode species, and the association of CT composition and larval motility in LFIA, were assessed with Pearson's correlations. The statistical significance was set at *P* < 0·05.

## RESULTS

### Plant fractions

The CT contents and structural characteristics of the 14 F2 fractions are presented in Table 1. The CT contents varied from 63·6 to 100 g CT/100 g fraction. The mDP ranged from 5·3 to 12·7 and the percentages of different tannin types covered almost the full range: 1–99% of PD and 4·3–95·6% of *cis-*stereochemistry within CT. The analyses of the correlations between these parameters showed non-significant low to moderate correlations: mDP *vs* PD (*r* = 0·4; *P* = 0·15), mDP *vs cis* (*r* = 0·21; *P* = 0·47) and PD *vs cis* (*r* = −0·34; *P* = 0·24). Additionally, we included the percentages of the different types of monomeric subunits within the CT fractions (C, EC, GC and EGC). The correlations with mDP were: low for EC (*r* = −0·12; *P* = 0·68) and GC (*r* = 0·20; *P* = 0·50) and moderate for C (*r* = −0·47; *P* = 0·09) and EGC (*r* = 0·45; *P* = 0·11). The correlations between subunits were more variable, and significant for EC *vs* GC (*r* = −0·68; *P* = 0·007). The characteristics of F1 fractions tested in the additional LFIA were: sainfoin (CT% = 37·2; mDP = 2·8; PD% = 72; *cis*% = 66·7), goat willow leaves (CT% = 51·5; mDP = 2·1; PD% = 5·8; *cis*% = 6·8) and pine bark (CT% = 54·0; mDP = 2·3; PD% = 15·1; *cis*% = 48·1).

**Table 1.** Plant sources and composition of purified condensed tannin (CT) fractions in terms of content (g CT/100 g fraction), mean degree of polymerization (mDP), percentages of procyanidins (PC), prodelphinidins (PD), *cis*- and *trans*-flavan-3-ols and molar percentages of monomeric subunits of CT [PC subunits: catechin (C) and epicatechin (EC); and PD subunits: gallocatechin (GC), epigallocatechin (EGC)]

Plant source	Botanical name	CT	mDP	PC	PD	*cis*	*trans*	GC	EGC	C	EC
Tilia flowers	*Tilia* spp.	91·7	7·9	99·1	0·9	95·6	4·4	0·0	0·9	4·4	94·7
Goat willow leaves	*Salix caprea*	83·8	5·3	95·2	4·8	4·3	95·8	3·0	2·0	92·7	2·3
Willow bark	*Salix* spp.	83·3	9·9	94·0	6·0	78·1	21·9	0·9	5·1	21·0	73·0
Pine bark	*Pinus* spp.	80·0	6·6	88·8	11·2	78·1	21·9	4·9	6·9	17·1	71·1
Hazelnut skin	*Corylus avellana*	67·5	9·1	79·1	20·9	47·8	52·2	12·2	9·6	40·1	38·1
Goat willow twigs	*Salix caprea*	93·2	5·3	78·7	21·3	62·8	37·2	9·5	12·7	27·8	50·0
Walnut leaves	*Juglans regia*	69·0	12·3	69·1	30·9	76·3	23·7	7·0	23·9	16·7	52·4
Weeping willow catkins	*Salix babylonica*	97·4	8·0	67·1	33·0	57·7	42·3	17·4	16·7	25·0	40·8
Birch leaves	*Betula* spp.	63·6	8·3	41·1	58·9	70·7	29·3	15·3	43·6	14·0	27·1
Sainfoin	*Onobrychis viciifolia*	100·0	8·7	35·2	64·9	79·2	20·9	14·8	51·3	6·1	27·8
Blackcurrant leaves A	*Ribes nigrum*	100·0	6·5	5·6	94·5	7·0	93·0	88·8	5·9	4·2	1·0
Blackcurrant leaves B	*Ribes nigrum*	77·1	11·8	4·7	95·3	18·8	81·2	77·9	17·4	3·3	1·4
White clover flowers A	*Trifolium repens*	100·0	8·6	1·3	98·7	58·9	41·1	40·5	58·3	0·7	0·5
White clover flowers B	*Trifolium repens*	82·4	12·7	1·2	98·8	61·8	38·2	37·8	61·0	0·3	0·8

### *In vitro* assays with first-stage larvae

#### Flavan-3-ol monomers

**Fig. 1.**
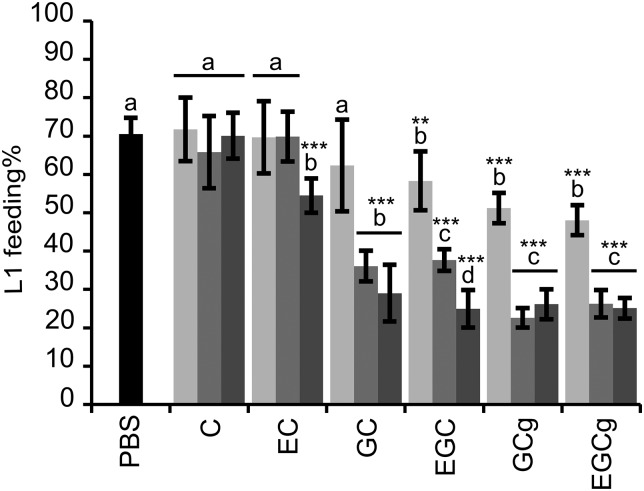
Results on the LFIA with flavan-3-ol monomers on mixed first-stage larvae (L1) (*Cooperia oncophora* and *Ostertagia ostertagi* 70:30). Bars represent the mean of fed larvae (%) from three replicates incubated in PBS (negative control) or pure flavan-3-ol monomers at 10 (light grey), 40 (grey) and 160 (dark grey) *μ*g mL^−1^ with error bars as s.d. Tested compounds were catechin (C) and epicatechin (EC) (i.e. procyanidin subunits), and gallocatechin (GC) and epigallocatechin (EGC) (i.e. prodelphinidin subunits) and their galloylated derivatives: gallocatechin gallate (GCg) and epigallocatechin gallate (EGCg). Significant differences between the mean in PBS and mean of each monomer at different dose levels are indicated by letters (asterisks **: *P* < 0·01; ***: *P* < 0·001). Thus, for each flavan-3-ol monomer different letters indicate statistical difference between doses (*P* < 0·05). Chemical structures of flavan-3-ol monomers are available from Brunet and Hoste (2006).

The flavan-3-ols that occur in PD tannins (GC and EGC) and their galloyl derivatives (GCg and EGCg) showed markedly higher reduction of L1 feeding ability as compared with the flavan-3-ols (C and EC) found in PC tannins (Fig. 1); whereas the other structural parameters, i.e. stereochemistry and galloylation, showed less pronounced effects on anthelmintic activity. The interaction between flavan-3-ol monomers and dose was significant (*P* < 0·001), and therefore, the estimates were calculated to compare the larval feeding inhibition for (i) dose effects for each flavan-3-ol monomer (Fig. 1) and (ii) flavan-3-ol monomers at each of the three dose levels. The dose-effect was significant for all flavan-3-ol monomers except for C. It was shown that presence of gallate in flavan-3-ols (GCg and EGCg) only slightly reduced the larval feeding at 40 *µ*g mL^−1^ in comparison with their respective non-galloylated flavan-3-ols (GC and EGC) (*P* < 0·05), and no difference was seen at other concentrations (*P* > 0·25). Moreover, an effect of *cis/trans* configuration was observed only between flavan-3-ols found in PC tannins. In fact, the *cis*-configured monomer (EC) inhibited larval feeding at 160 *µ*g mL^−1^ [*P* < 0·001; and *P* < 0·01 for comparisons with PBS (=negative control) and C at 160 *µ*g mL^−1^, respectively], while the *trans*-configuration (C) showed no effect at any of the concentrations as compared with the negative control.

Likewise, the motility of L1 was substantially reduced with flavan-3-ols found in PD compared with those found in PC tannins. At 40 and 160 *µ*g mL^−1^ no L1 motility was observed when exposed to GC, EGC, GCg or EGCg while flavan-3-ols C and EC did not affect the larval motility, not even at high concentrations, as compared with the negative control (brief observation without counting).

#### CT fractions

**Fig. 2.**
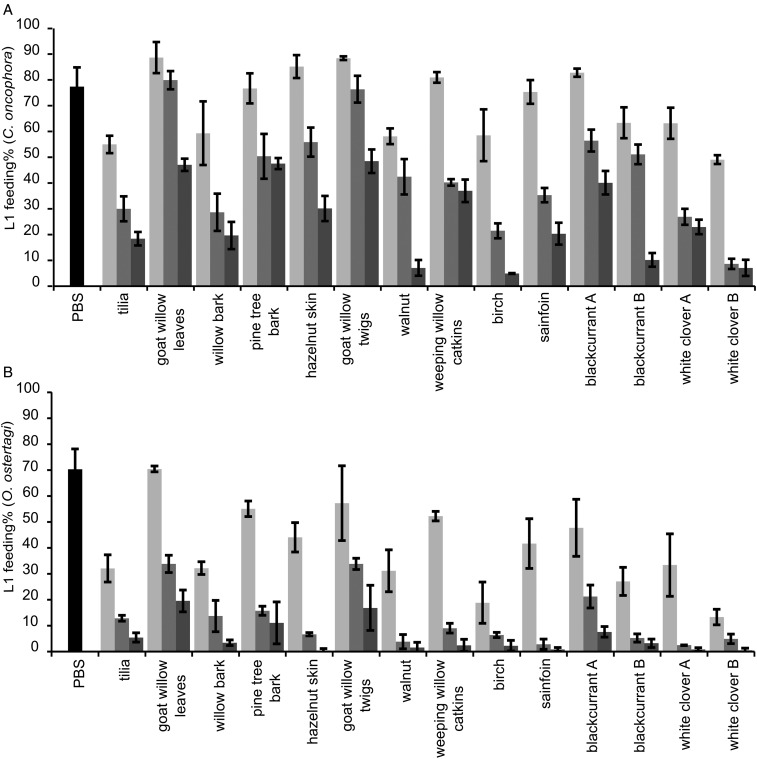
Results of the LFIA with 14 CT fractions on L1 of *Cooperia oncophora* (A) and *Ostertagia ostertagi* (B). Bars represent the mean percentage of fed larvae (%) from three replicates for each fractions at 2·5 (light grey), 10 (grey) and 40 (dark grey) *μ*g of CT mL^−1^ and the pooled mean for the negative control (PBS) with error bars as s.d. The chemical structure of CT can be found in (Williams *et al.* 2014*a*).

**Fig. 3.**
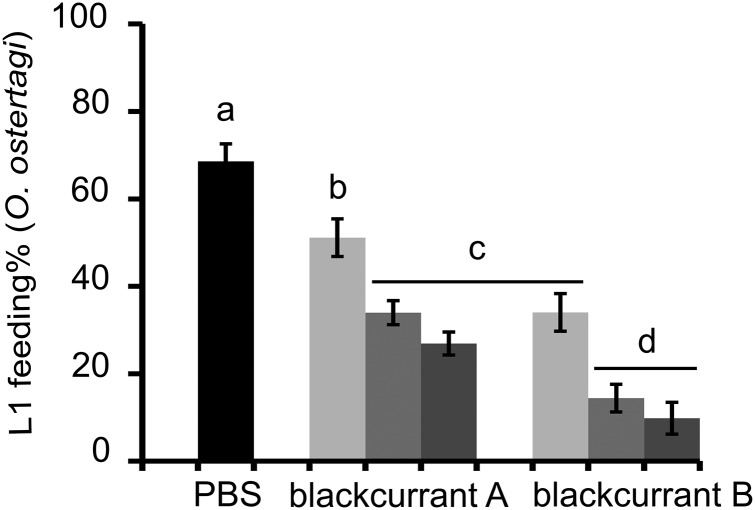
Results of a LFIA with *O. ostertagi* L1, modified in order to avoid interaction between CT and the bacterial food source. Bars represent the mean percentage of fed larvae incubated in PBS (negative control) or CT fractions at 2·5 (light grey), 10 (grey) or 40 (dark grey) *μ*g of CT mL^−1^ with error bars as s.d.. Different letters indicate a significant difference (one-way ANOVA with Tukey’ HSD *post hoc* test; *P* < 0·05).

While significant dose dependent interactions were observed for CT (*P* < 0·01) each parameter was statistically assessed at each concentration. Overall, CT fractions showed a dose-dependent reduction of the larval feeding, which was higher than their respective flavan-3-ol monomers at the same w/v concentrations (Fig. 2). This was substantiated by a statistically significant negative effect of mDP at all concentrations on the larval feeding (*P* < 0·01). Moreover, the feeding of *O. ostertagi* L1 in the modified LFIA was significantly reduced with the CT fraction that had the higher mDP (Fig. 3), in the same manner as LFIA following the standard protocol. The motility of L1 was also strongly reduced with both CT fractions as compared with PBS. The higher percentages of *cis* and PD subunits also reduced, but to a lesser extent, the larval feeding ability (Fig. 2). These effects were statistically significant only at 2·5 and 10 *µ*g of CT mL^−1^ for *cis* (*P* < 0·01) and at 10 *µ*g of CT mL^−1^ for PD subunits (*P* < 0·05). Moreover, these variables were kept in the model as they did not show problematic collinearities (VIF < 2). The analysis based on CT subunits was abandoned as it showed strong collinearities and confounding effects (VIF > 1000).

Non-CT compounds (=100% – CT percentage) for each fraction were excluded from the statistical model as they did not show any significant effect (Note: fraction concentrations in the assay were adjusted to account for their varying CT contents). Results of the supplementary LFIA with F1 fractions, adjusted for CT content, showed a poor inhibition for sainfoin and goat willow leaves as compared with pine bark (mean inhibition ± s.d.: 16·1% ± 0·5, 13·2% ± 2·5 and 31·6% ± 3·5, respectively). The pre-incubation of sainfoin F2 with or without sucrose had no effect on the LFIA (similar results as shown in Fig. 2B).

The larval feeding in the control media was lower for *O. ostertagi* compared with *C. oncophora*, although not statistically significant. However, the results with CT indicated that L1 of *O. ostertagi* were more susceptible to CT than *C. oncophora*: at all three CT concentrations the L1 feeding was much lower for *O. ostertagi* (*P* < 0·001). In addition, the correlations between the mean percentages of larval feeding inhibition for the two nematode species were significant at all concentrations (*P* < 0·01) and overall (*P* < 0·001).

**Fig. 4.**
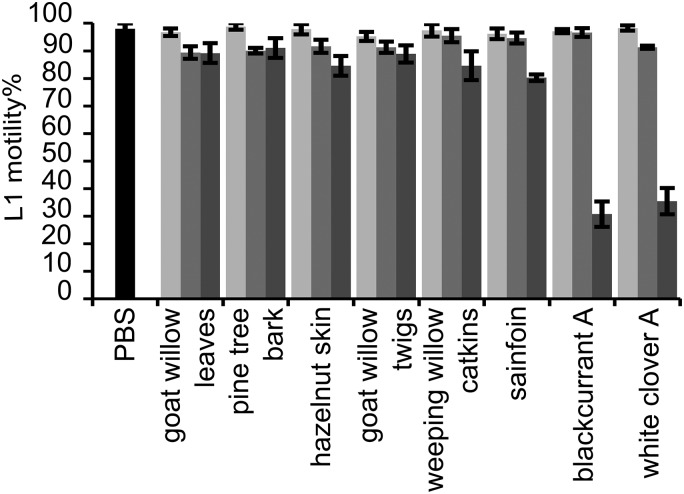
Motility of the first-stage larvae (L1) of *Ostertagia ostertagi* in the presence of eight different CT fractions. Experiment was performed at the same time as the larval feeding (see Fig. 2B). Any larval movement within 5 s was counted as motile. Bars represent either the pooled mean for the negative control (PBS) or the mean of the percentage of motile L1 incubated at 2·5 (light grey), 10 (grey) and 40 (dark grey) *μ*g of CT mL^−1^ in triplicates with error bars as s.d.

The motility of *O. ostertagi* L1 was reduced in a dose-dependent manner with most of the CT fractions, which could have been more prominent at lower CT concentrations by using a scaled score instead of a dichotomous scale. Furthermore, the motility was clearly inhibited at 40 *µ*g of CT mL^−1^ with PD-rich fractions (white clover A and blackcurrant A) (Fig. 4) and was similar to the effects of flavan-3-ol monomers. In fact, the few remaining motile larvae with these two CT fractions were only moving very slowly. Thus, the percentage of PD was the main factor responsible for the higher inhibition of the larval motility at 40 *µ*g mL^−1^ (*r* = −0·92; *P* = 0·001) and no other structural parameter was statistically correlated with inhibition of larval motility.

### *In vitro* assay with CT fractions and adult worms

**Fig. 5.**
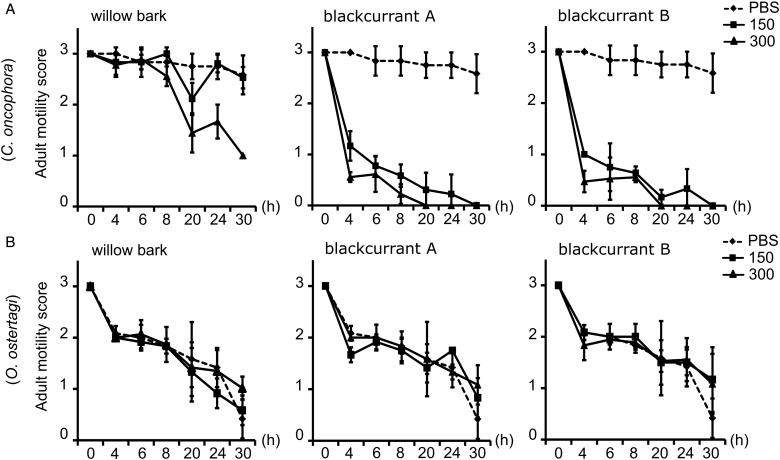
Results on the motility of adult worms of *Cooperia oncophora* in row (A) and *Ostertagia ostertagi* in row (B), incubated in triplicates either in control media (PBS) or CT fractions of willow bark or blackcurrant leaves at 150 and 300 *µ*g mL^−1^. The motility was assessed within 10 s by observation using a scaled score 0–3: with 3 (active movement), 2 (slow movement), 1 (only moving one part of the body) and 0 (no movement). At each time point the average motility score of the triplicates was plotted with error bars showing the s.d.

The three CT fractions inhibited the motility of *C. oncophora* adults, although to different extents, as compared with adult worms in the control media, which were actively moving throughout the observation period (30 h). The two CT fractions from blackcurrant leaves, that contained mainly GC but differed in mDP, showed higher anti-parasitic potency when compared with the CT fraction from willow bark which contained mainly (EC) (Fig. 5A). The motility was greatly reduced after 4 h with 150 and 300 *µ*g mL^−1^ of PD-rich CT fractions, and completely inhibited with these two concentrations after 20 and 30 h, respectively. In comparison, CT from willow bark at 150 *µ*g mL^−1^ did not change the nematode motility as compared with the control. Moreover, 300 *µ*g mL^−1^ reduced the motility only after 20 h, and this concentration was not sufficient to kill the worms even after 30 h.

**Fig. 6.**
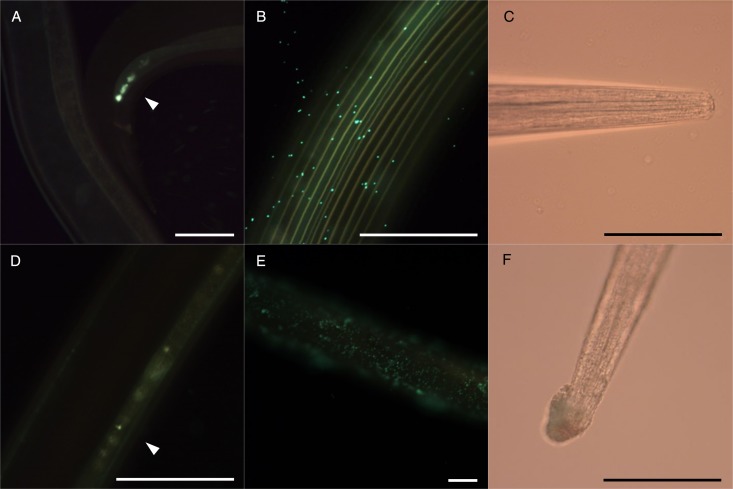
Micrographs of *Ostertagia ostertagi* adults pre-incubated in control media (A, B, C) or CT fraction from blackcurrant leaves A at 300 *µ*g mL^−1^ (D, E, F), for 2 h (A, D) or 30 h (B, C, E, F), and then transferred to control media containing fluorescent *E. coli* for 24 h. White arrow heads show presence of fluorescent bacteria in the cloacae (A) or in the digestive tract (B) of the worm. (B, E) show a part of the cuticle and (C, F) the anterior part (scale bars = 100 *µ*m).

**Fig. 7.**
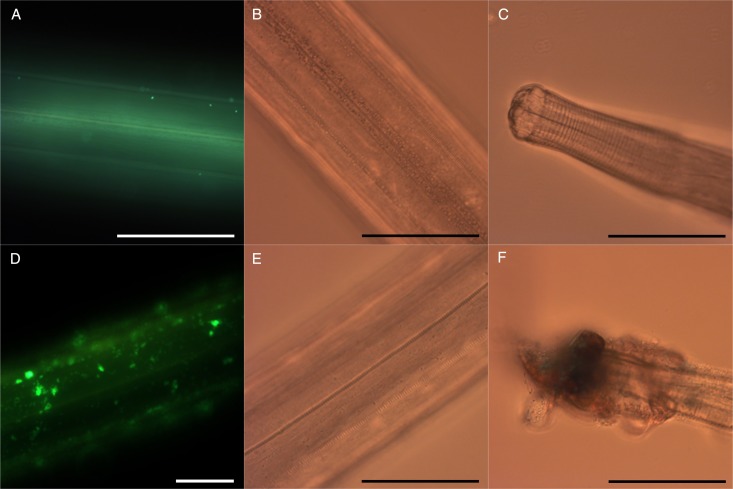
Micrographs of *Cooperia oncophora* adults pre-incubated for 5 days at 38 °C in control media (A, B, C) or CT fraction from blackcurrant leaves at 300 *µ*g mL^−1^ (D, E, F), and then transferred to control media containing fluorescent *E. coli* for 24 h. The cuticle is shown by fluorescent microscopy (A, D) or light microscopy (B, E) and of the anterior part is illustrated in (C, F) (scale bars = 100 *µ*m).

In contrast, adult *O. ostertagi* worms had a reduced motility and died within 30 h in the control media (Fig. 5B), although worms were very active at the time of allocation to the wells. Moreover, at both concentrations no CT fractions showed any difference as compared with the negative control. Although the fitness of adults of *O. ostertagi* was weak during the whole incubation period, the majority of worms were still alive after 20 h with PD-rich CT fractions at 300 *µ*g mL^−1^ (unlike *C. oncophora* adults). Hence, we attempted to verify whether *O. ostertagi* worms were deprived of their ability to feed and subsequently unable to ingest CT. We used a method similar to the LFIA. Briefly, we incubated a few *O. ostertagi* adults in one well with control media only or with CT fractions from willow bark and blackcurrant leaves (samples A and B) at 300 *µ*g mL^−1^ for 2 or 30 h at 38 °C. Then, the worms were transferred individually into a well-holding fresh control media containing fluorescent *E. coli*, and incubated for 24 h at 38 °C. The worms were then individually washed in PBS before visualization by microscopy at 400×. We observed traces of fluorescent *E. coli* in the pharyngeal region or the digestive tract of most of the worms pre-incubated for 2 h with or without CT (Fig. 6A and D). Moreover, the pumping activity of the pharyngeal muscles was also observed in a few live worms but the feeding behaviour could not be compared with *C. oncophora* adult worms as the assays with this species were done 1 week earlier. Additionally, we could see the main structures of the external surface of the worms at the highest magnification, even in the presence of fluorescence. The longitudinal lines of the external cuticle of *O. ostertagi* adults appeared smooth in all groups after 2 and 30 h incubations with or without CT (Fig. 6B and E). However, aggregates around the anterior part were seen only on worms incubated with CT for 2 h and were more pronounced after 30 h (Fig. 6F). Moreover, the cuticle was also covered with aggregates and fluorescent *E. coli* for the worms pre-incubated for 30 h in CT (Fig. 6E), but not the worms incubated in control media (Fig. 6B) or in CT fractions for 2 h. Similar pictures were obtained from dead *C. oncophora* adult worms that had been incubated in the same control media and CT for 5 days (Fig. 7). The surface of the cuticle was not obviously damaged, although aggregates were observed on the cuticle and the anterior part of the worms, similarly to *O. ostertagi*.

## DISCUSSION

It is evident from our findings that the CT levels are decisive for the anti-parasitic activity. This was reflected by a dose-dependent reduction in L1 feeding for all CT fractions in the LFIA, although to different extents. A dose response effect in the LFIA was also found for most flavan-3-ol monomers. Our results also showed that mDP was the main structural CT parameter that influenced the inhibition of feeding without affecting the motility. Motility was instead strongly related with a high percentage of PD for both L1 and adult worms. This is the first report of how these two structural parameters of CT exert different anthelmintic effects. It also clearly shows that the main effect of one parameter may complicate the analysis of effects of other structural parameters. Apparently, the CT stereochemistry (*cis*- or *trans-*configurations) had only a minor effect on the anti-parasitic properties as compared with the two other parameters.

We found that *O. ostertagi* L1 were more susceptible to CT than *C. oncophora* L1, in contrast to previous studies that did not detect any species-specific differences (Novobilský *et al.* 2011, 2013). Moreover, the control values in the LFIAs ranged between 75 and 80%, in the same manner as previously reported (Novobilský *et al.* 2013) or slightly higher (Novobilský *et al.* 2011; Peña-Espinoza *et al.* 2015). One explanation for the different susceptibilities could be that different *O. ostertagi* strains were used. However, several *in vitro* studies with sheep GIN have also reported a greater susceptibility of abomasal nematodes to CT (e.g., *H. contortus, Teladorsagia circumcincta*) as compared with intestinal nematodes (e.g., *T. colubriformis*). This has been demonstrated for different life stages in different assays: egg hatchability, larval development (eggs to L3), migration of L3 (Molan *et al.* 2004; Molan, 2014) and L3 exsheathment (Quijada *et al.* 2015). Further, the inhibition of larval feeding by the different CT fractions was significantly correlated for both nematode species, indicating that the CT structure influenced the anthelmintic activity similarly for both species, in this assay. In fact, the species differences with regards to the CT structure were minor in studies that targeted the exsheathment of L3 in small ruminants (Brunet and Hoste, 2006; Quijada *et al.* 2015).

The influence of mDP on CT-induced reduction of larval feeding is in agreement with a previous study by Novobilský *et al.* (2013), although the present study explored a substantially smaller range of mDP values, i.e. 5–13 *vs* 2–95. In fact, this effect proved to be the most important for larval feeding, among other CT structure traits. Moreover, we showed that this effect was targeted directly against the larvae and not due to an interference with *E. coli* (Fig. 3). In fact, larger CT have been shown to have a greater binding capacity to bacterial surfaces which could have modified the feed source in the LFIA, perhaps by aggregation (Jones *et al.* 1994; Verhelst *et al.* 2013). This could have rendered the bacteria less accessible thereby indirectly influencing the feeding ability of L1.

Higher PD and *cis*-subunit percentages in CT also reduced the larval feeding but to a lesser extent than mDP, as they were not statistically different at all concentrations. The CT stereochemistry had no significant effect in a previous study (Novobilský *et al.* 2013), most likely because the range was too narrow (60–82% *cis*-subunits in CT). The present set of experiments has now investigated a much wider range (5–96% *cis-*subunits) but was still only able to detect a few significant differences. A more detailed analysis based on the percentages of all CT subunits (C, EC, GC and EGC) was not possible because of strong collinearities and confounding effects. Therefore, it remains easier to interpret results with the commercial flavan-3-ol monomers and to distinguish their anthelmintic properties in relation to their structures. Thus, we have clearly shown that flavan-3-ols giving rise to PD (GC, EGC) were more effective in the LFIA than flavan-3-ols that are found in PC (C, EC); and the *cis*-configuration was of a less importance as EC only had a slightly stronger effect than C (whereas no statistical difference between EGC and GC was observed). Although these results are based on a mixture of nematode species, they are comparable with other studies that tested flavan-3-ols against the exsheathment of *H. contortus* L3 (Brunet and Hoste, 2006) and the motility of *A. suum* L4 (Williams *et al.* 2014*a*). The galloylation of flavan-3-ols also seemed to enhance the anthelmintic activity in the LFIA, as previously reported for L3 in sheep (Molan *et al.* 2003; Brunet and Hoste, 2006). However, we did not test the gallate-derivatives of flavan-3-ols that occur in PC (Cg or ECg) and thus, could not perform a full comparison.

For first stage larvae of both *O. ostertagi* and *C. oncophora* the motility was markedly reduced only with PD-rich CT fractions, GC and EGC, and their galloyl derivatives (GCg, EGCg) at ⩾40 *µ*g mL^−1^. This was strongly supported by the results of the adult motility assay with *C. oncophora*, where CT fractions from blackcurrant leaves (PD-rich) were distinctly more potent than those of willow bark (PC-rich) regardless of their mDP values. Although we did not test the influence of mDP with PC-rich fractions in the adult motility assay, this is expected to be minor compared to the overriding effect of the PD percentage. In fact, Spiegler *et al.* (2015) have recently shown that a degree of polymerization (DP) of 3 in PC oligomers represented the lower threshold that significantly reduced the survival of adult *Caenorhabditis elegans*, although to a limited extent (maximum reduction of approximately 55%), and a DP > 4 had no further effect on the anthelmintic activity.

Our findings highlight differences between CT effects on feeding and motility. This can perhaps be explained by various modes and sites of CT action. With the feeding it is expected that larger CT have prevented the ingestion of *E. coli* to a high degree by obstruction of the mouth of the larvae or by precipitation of proteins in the buccal cavity. Precipitates probably confer a pronounced astringency, even at very low CT concentrations, thereby depriving larvae of energy, impairing digestive processes and slowing or blocking development. In fact, ellagitannins do reduce the development of *C. elegans* L1 (Mori *et al.* 2000), and CT have been shown *in vitro* to be detrimental for the moulting of *O. dentatum* L3 to L4 in pigs (Williams *et al.* 2014*b*). Motility was not affected in any of these studies. Likewise, the majority of *O. ostertagi* L1 that we exposed to 10 *µ*g of CT mL^−1^ of fractions was unfed (Fig. 2B) but remained fully motile (Fig. 4). The lack of feeding is expected to be detrimental to larval development which may be interpreted as an early manifestation of CT toxicity; we did, however, not follow the effect on larval development over time. Then, the reduced motility of L1 incubated with PD-rich fractions at 40 *µ*g of CT mL^−1^ relative to PC-rich fractions was not related to starvation but rather to some other mechanism. Williams *et al.* (2014*a*) have shown that *A. suum* L4 incubated with CT fraction from hazelnut skin (PC-rich) were motionless after 12–24 h, which was related with substantial structural damage on external and internal tissues of the larvae. Moreover, the increasing percentage of PD subunits in CT has been often associated with a greater anthelmintic activity (Novobilský *et al.* 2013; Quijada *et al.* 2015) as in our study; yet, it has never been shown whether PC and PD-rich CT induce structural damage of the worms to a different extent.

The low survival of adult *O. ostertagi* as compared with *C. oncophora* in our control medium in the adult motility assay prevented comparison of the two species. A similar observation was made earlier when the motility of adult *H. contortus* was tested against *T. colubriformis* (Paolini *et al.* 2004). In our specific case, this finding could have been influenced by a suboptimal medium and/or use of older worms. In fact, Geldhof *et al.* (2000) used 21-day-old adult *O. ostertagi*, which were kept alive for at least 48 h in supplemented RPMI medium whereas our worms were 38-day-old and died after 24 h in PBS. Moreover, the motility of these worms was not reduced by CT in contrast to *C. oncophora* where an effect could be observed 4 h post-incubation. Mori *et al.* (2000) concluded that the reduced survival of young *C. elegans* adults exposed to ellagitannins was likely related to the ingestion of compounds as the cuticle of the worms was not visibly damaged. In our study, the changes on the surface of adult worms exposed to CT were mainly characterized by aggregates around the mouth and on the cuticle while pronounced folds on the cuticular ridges, as observed in adult *H. contortus* (Martínez-Ortíz-de-Montellano *et al.* 2013), were not seen. The lesions were comparable between the two cattle nematode species and could evidently not account for the difference in motility observed between the species. We observed that adult *O. ostertagi* were able to ingest *E. coli* to some extent; however, the uptake of CT probably remained too low to observe any effect. This emphasizes the importance of the contact route with CT on their anthelmintic activity.

Although the presence of non-CT compounds in the CT fractions had no statistically significant effect on the LFIA, it may have influenced some of the results. It could perhaps explain the lower larval feeding in the presence of birch leaf CT that had the lowest CT content as compared with sainfoin tannins, despite their close structural similarities. Additionally, we obtained a poor inhibition in LFIA with the F1 fraction from sainfoin with lower mDP and CT content values as reported previously for the motility of *A. suum* L3 (Williams *et al.* 2014*a*). This could not be explained by the interference of impurities such as sugars, as the pre-incubation of CT with sucrose had no effect on anthelmintic activity; thus, confirming findings by Williams *et al.* (2014*a*). Nonetheless, it has been shown that the disruptive effect of carbohydrates on the formation of protein–tannin complexes depends on the type of carbohydrate and, more importantly, decreases with larger CT (Mateus *et al.* 2004). Therefore, the putative interaction of sugars with CT that is expected to reduce the anthelmintic activity may have been stronger with low mDP tannins (F1 fractions) as compared with higher mDP tannins (F2 fractions) from sainfoin.

In conclusion, CT showed activity against free-living larvae and parasitic adults of *O. ostertagi* and *C. oncophora*, confirming the potential role of these bioactive compounds in control of GIN in cattle. L1 of *O. ostertagi* were more susceptible to CT than *C. oncophora*, but the influence of the CT structure on the anthelmintic activity was similar for both species. Hence, the reduction of nematode motility was highly influenced by the PD percentage in CT, whereas tannin size (in terms of mDP-values) was of importance only when CT interfered with larval feeding ability. Further research is needed to investigate the targets of CT on the cuticle and the digestive tract of parasitic nematodes and thus their exact mechanisms of action.
